# Supplementary material for: Delayed tumor-draining lymph node irradiation preserves the efficacy of combined radiotherapy and immune checkpoint blockade in models of metastatic disease
Source: Nat Commun. 2024 Jun 29;15:5500. doi: 10.1038/s41467-024-49873-y (PMC11217506; doi:10.1038/s41467-024-49873-y)
Supplement: Supplementary file 1 — Supplementary Information [file 41467_2024_49873_MOESM1_ESM.pdf]

Supplementary Information

Delayed Tumor-Draining Lymph Node Irradiation Preserves the Efficacy of Combined Radiotherapy and Immune Checkpoint Blockade in Models of Metastatic Disease

Irma Telarovic, Carmen S.M. Yong, Lisa Kurz, Irene Vetrugno, Sabrina Reichl, Alba Sanchez Fernandez, Hung-Wei Cheng, Rona Winkler, Matthias Guckenberger, Anja Kipar, Burkhard Ludewig, Martin Pruschy

Contents

Supplementary Figure 1. Changes in the tumor immune cell composition following draining lymph node irradiation.

Supplementary Figure 2. Adjuvant draining lymph node irradiation improves regional lymph node control, mitigates the growth of a distant (non-irradiated) tumor and allows for the induction of long-lasting tumor-specific immunity.

Supplementary Figure 3. Adjuvant draining lymph node irradiation improves the treatment response also in the murine MC38 colon carcinoma model.

Supplementary Figure 4. Changes in the draining lymph node immune cell composition following irradiation.

Supplementary Figure 5. Representative sections of irradiated lymph nodes.

Supplementary Figure 6. Irradiation induces changes in the stromal cell compartment of the lymph node and interferes with the CCR7-CCL19/CCL21 immune cell homing axis.

Supplementary Figure 7. Representative flow cytometry gating strategy for the analysis of DCs.

Supplementary Table 1. Antibodies used for flow cytometry and fluorescence activated cell sorting.

Supplementary Table 2. Antibodies used for immunofluorescence.

Supplementary Figure 1

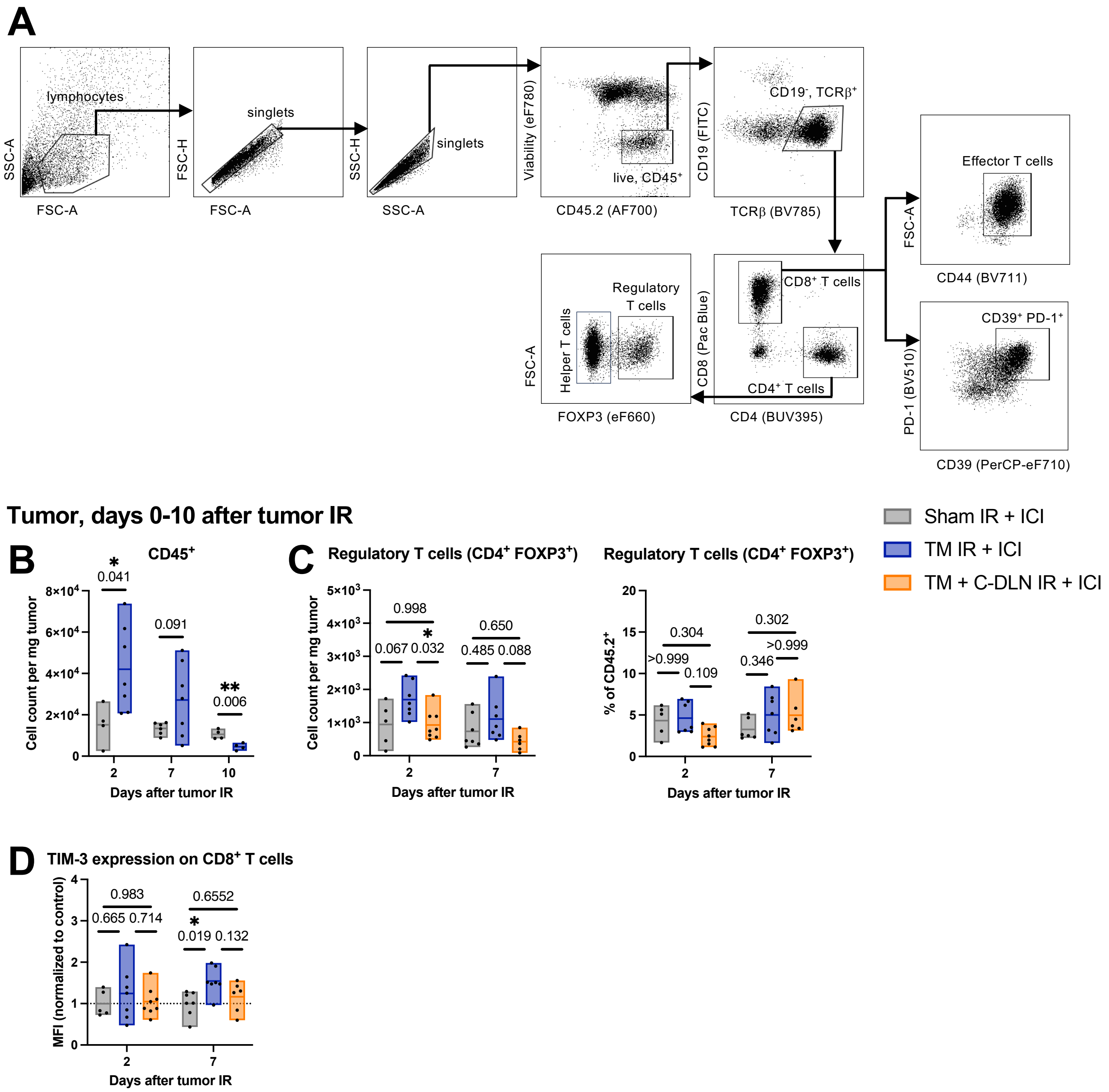

Supplementary Figure 1. Changes in the tumor immune cell composition following draining lymph node irradiation. (A) Representative gating strategy for tumor immunophenotyping. Corresponding data is shown in Figure 3, D to H, Figure 4G, and Supplementary Figure 1, B to D. **(B to D)** Immunophenotype of the tumor over 10 days after tumor IR, as described in Figure 3. All groups received α-CTLA-4. "Sham IR + ICI" group (gray) received sham IR, "TM IR + ICI" group (blue) received tumor IR, and "TM + C-DLN IR + ICI" group (orange) received DLN IR concomitantly to tumor IR. Each dot represents an individual mouse. Floating bars span from the minimal to the maximal value of each group. Line indicates the mean. $n \geq 4$ mice per group (exact numbers provided in Source Data file). (B) $CD45^+$ cell count per mg tumor. (C) Tumor-infiltrating regulatory T cells. Left: Cell count per mg tumor. Right: Percentage of $CD45^+$ cells. (D) TIM-3 expression on $CD8^+$ T cells on days 2 and 7 after tumor IR, expressed as the geometric mean of the fluorescence intensity (MFI), normalized to the average MFI value of the "Sham IR + ICI" group. Data were tested for normality using the Shapiro-Wilk test. For data following a normal distribution, treatment groups were compared using the two-sided unpaired t test (B) or one-way ANOVA with Holm-Sidak's multiple comparisons test (C, left and D). For non-normally distributed data, the comparison was performed using the Kruskal-Wallis test with Dunn's multiple comparisons test (C, right). All p values are displayed, with *, ** and *** indicating $p<0.05$, $p<0.01$ and $p<0.001$, respectively.

Supplementary Figure 2

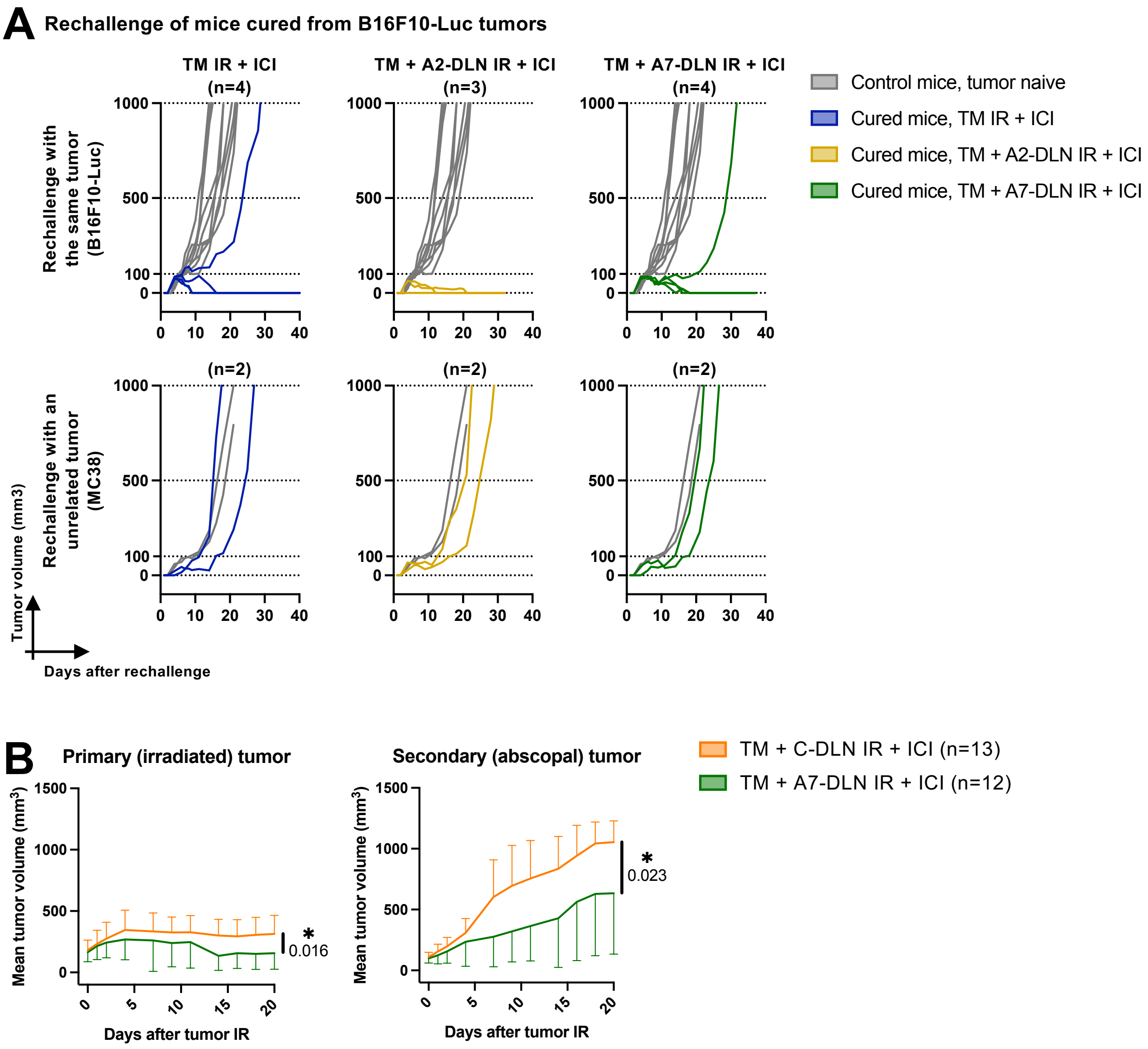

Supplementary Figure 2. Adjuvant draining lymph node irradiation improves regional lymph node control, mitigates the growth of a distant (non-irradiated) tumor and allows for the induction of long-lasting tumor-specific immunity. (A) Individual tumor growth curves in response to the rechallenge with the same (B16F10-Luc, left) or an antigenically unrelated (MC38, right) tumor cell line. Complete responders are shown in blue, yellow and green for "TM IR + ICI", "TM + A2-DLN IR + ICI" and "TM + A7-DLN IR + ICI" group, respectively. Tumor-naïve control mice are shown in gray. Each line represents an individual mouse. Number of cured mice in each group is indicated in the corresponding graph title. For the naïve control mice, n=8 for the B16F10-Luc group and n=2 for the MC38 group. **(B)** Mean tumor volume curves for primary and secondary tumors of the mice shown in Figure 6, D to H. Error bars represent the standard deviation (SD). Number of mice is indicated in the figure. p values are derived from the comparison of mean tumor volumes on day 20 after IR, using the two-sided Mann-Whitney test (due to the non-normal distribution of the data, as determined by the Shapiro-Wilk test). All p values are displayed, with *, ** and *** indicating $p<0.05$, $p<0.01$ and $p<0.001$, respectively.

Supplementary Figure 3

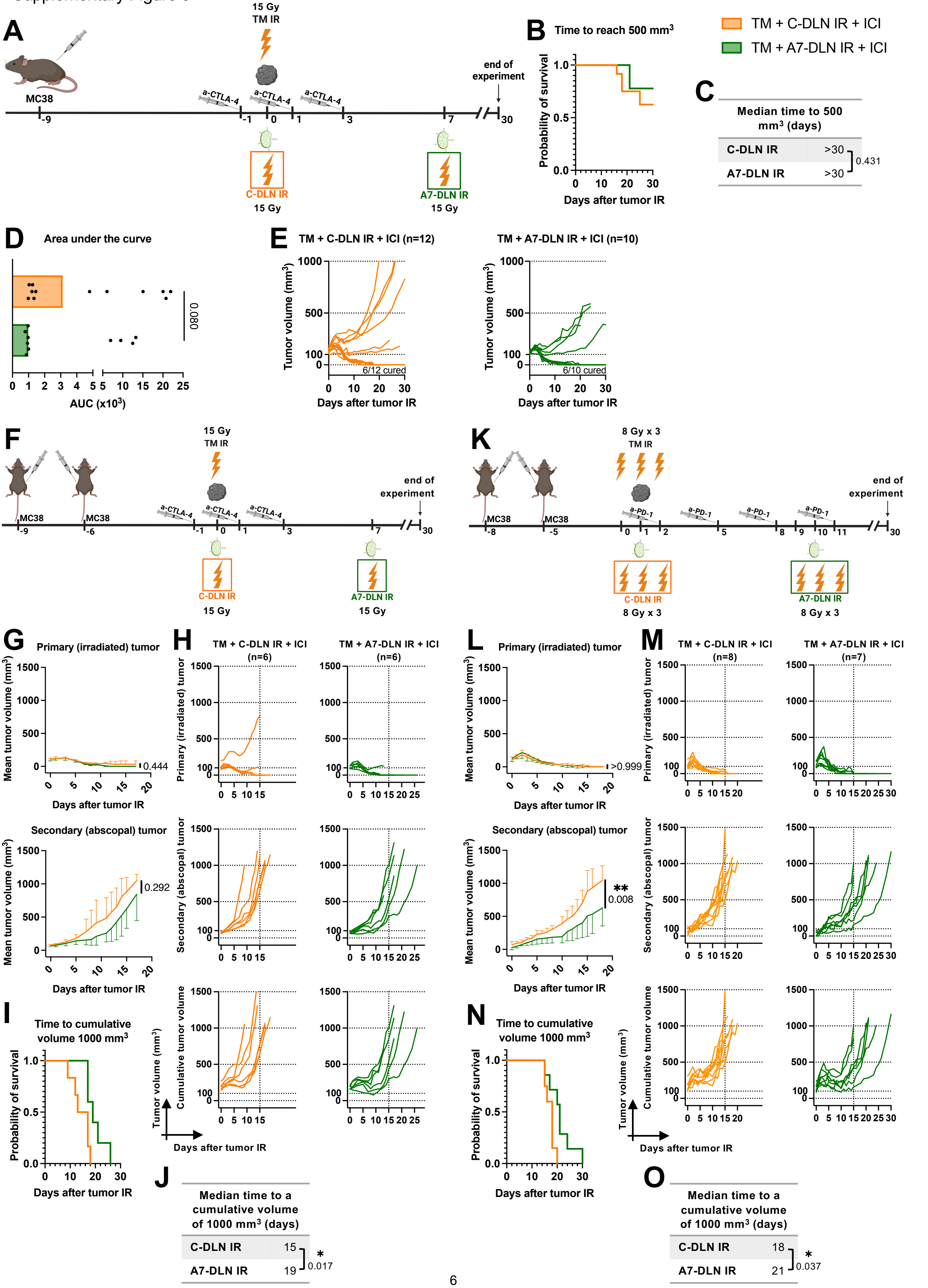

Supplementary Figure 3. Adjuvant draining lymph node irradiation improves the treatment response also in the murine MC38 colon carcinoma model. (A) Mice received tumor IR and α-CTLA-4, as indicated. DLN IR was performed either together with the tumor IR (C-DLN IR, orange), or 7 days thereafter (A7-DLN IR, green). **(B to E)** Treatment response represented by the Kaplan-Meier survival analysis (B and C), area under the curve (AUC) analysis (D) and individual tumor growth curves (E). Each dot in (D) and each line in (E) represents an individual mouse. Bar width in (D) represents the median value of the corresponding group. Number of mice is indicated in (E). **(F)** Mice received tumor IR and α-CTLA-4 as, indicated. DLN IR was performed either together with the tumor IR (C-DLN IR, orange), or 7 days thereafter (A7-DLN IR, green). **(G to J)** Treatment response represented by the mean tumor volume curves (G), individual tumor growth curves (H) and the Kaplan-Meier survival analysis (I and J)**. (K)** Mice received tumor IR, α-CTLA-4 and α-PD-1, as indicated. DLN IR was performed either together with the tumor IR (C-DLN IR, orange), or 7 days thereafter (A7-DLN IR, green). **(L to O)** Treatment response represented by the mean tumor volume curves (L), individual tumor growth curves (M) and the Kaplan-Meier survival analysis (N and O). Time to reach a cumulative tumor volume (i.e. the sum of the primary and secondary tumor volume on a given day) of 1000 mm^3 was used as the endpoint for Kaplan-Meier analysis. Error bars in (G) and (L) represent the standard deviation (SD). Each line in (H) and (M) represents an individual mouse. Number of mice is indicated in (H) and (M). Data were tested for normality using the Shapiro-Wilk test. For data following a normal distribution, treatment groups were compared using the two-sided unpaired t test (G, bottom and L, bottom). For non-normally distributed data, comparisons were performed using the two-sided Mann-Whitney test (D, G, top and L, top). Logrank test (Mantel-Cox) was used to compare the survival curves in (B), (I) and (N); corresponding p values are displayed in (C), (J) and (O), respectively. All p values are displayed, with *, ** and *** indicating $p<0.05$, $p<0.01$ and $p<0.001$, respectively. Supplementary Figure 3, panels A, F and K created with BioRender.com, released under a Creative Commons Attribution-NonCommercial-NoDerivs 4.0 International license.

Supplementary Figure 4

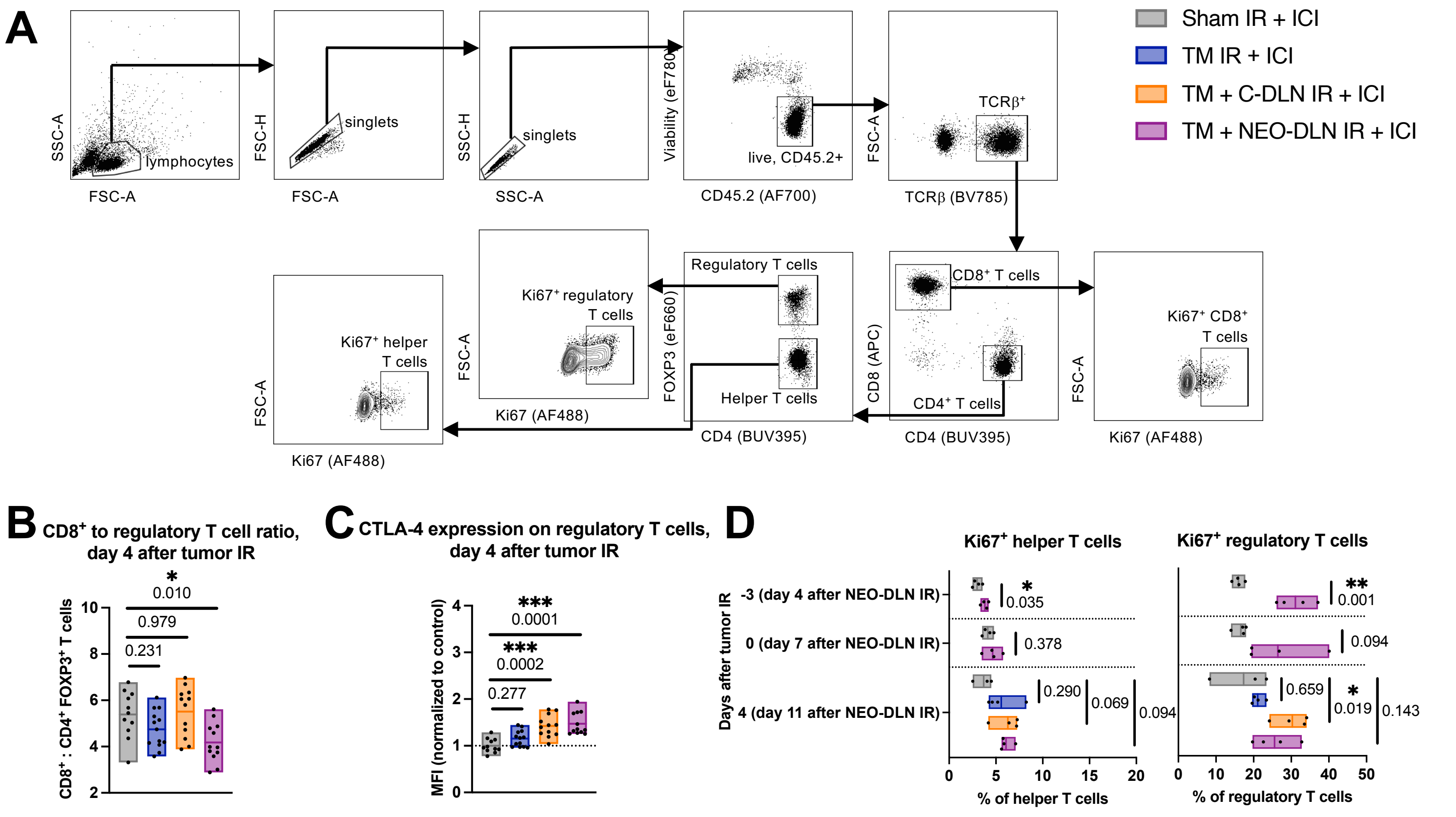

Supplementary Figure 4. Changes in the draining lymph node immune cell composition following irradiation. (A) Representative gating strategy for lymph node immunophenotyping. Corresponding data is shown in Figure 7, B to G, and Supplementary Figure 4, B to D. **(B to D)** Immune cell composition of the DLN in response to IR, as described in Figure 7. All groups received α-CTLA-4. "Sham IR + ICI" group (gray) received sham IR, "TM IR + ICI" group (blue) received tumor IR, "TM + C-DLN IR + ICI" group (orange) received DLN IR concomitantly to the tumor IR, and "TM + NEO-DLN IR + ICI" group (purple) received DLN IR 7 days prior to tumor IR. Each dot represents an individual mouse. Floating bars span from the minimal to the maximal value of each group. Line indicates the mean. n≥11 for (B) and (C), and n≥3 mice per group for (D) (exact numbers provided in Source Data file). (B) $CD8^+$ to regulatory T cell ratio in the DLNs on day 4 after tumor IR. (C) CTLA-4 expression on regulatory T cells on day 4 after tumor IR, expressed as the geometric mean of the fluorescence intensity (MFI), normalized to the average MFI value of the "Sham IR + ICI" group. (D) Percentage of $CD4^+$ $FOXP3^-$ helper T cells (left) and $CD4^+$ $FOXP3^+$ regulatory T cells (right) in the DLN positive for Ki67. Data were tested for normality using the Shapiro-Wilk test. For data following a normal distribution, treatment groups were compared using the two-sided unpaired t test (B and D, day -3) or one-way ANOVA with Holm-Sidak's multiple comparisons test (B and D, day 4). For non-normally distributed data, comparisons were performed using the two-sided Mann-Whitney test (D, day 0) or the Kruskal-Wallis test with Dunn's multiple comparisons test (C). All p values are displayed, with *, ** and *** indicating p<0.05, p<0.01 and p<0.001, respectively.

Supplementary Figure 5

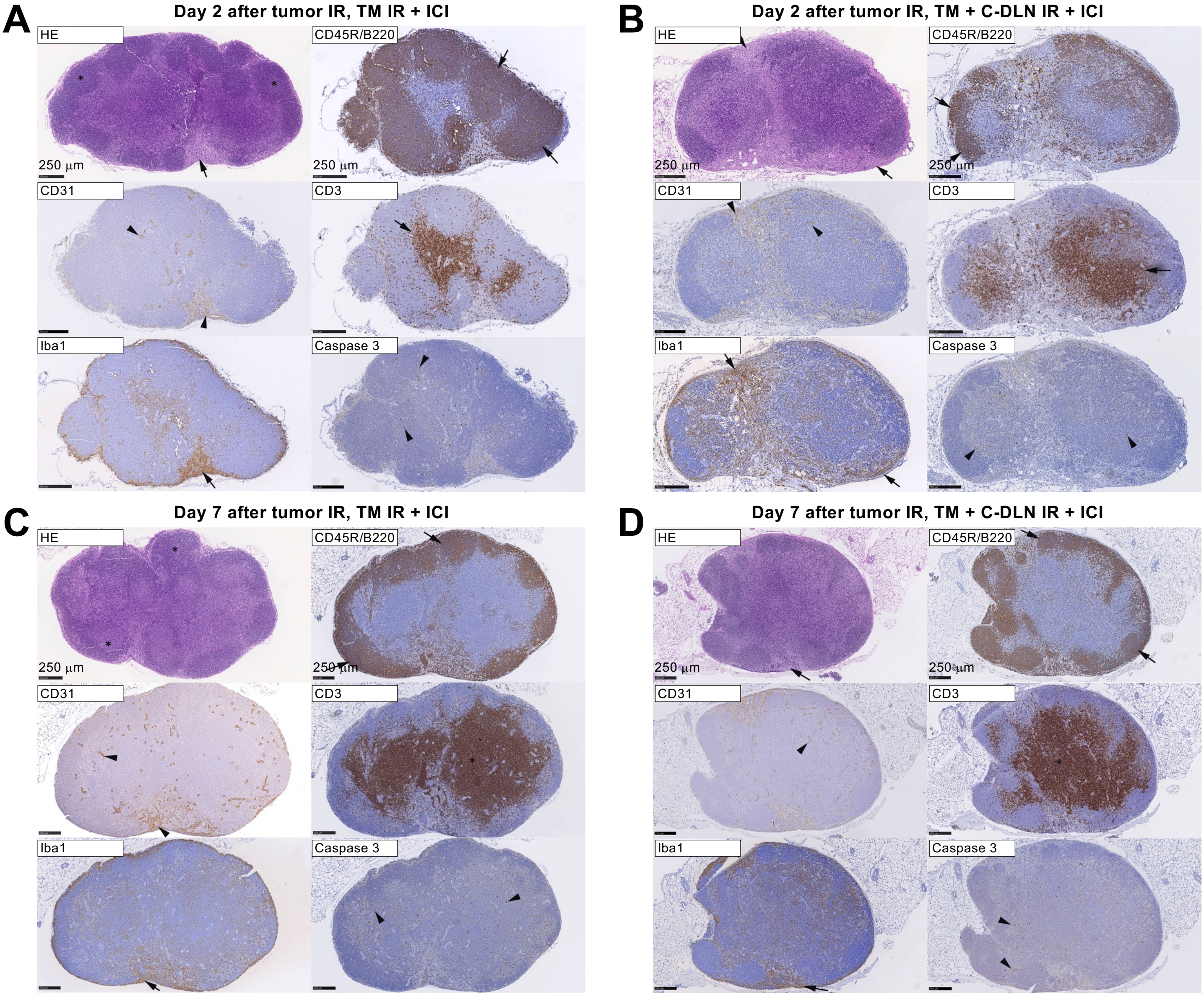

Supplementary Figure 5. Representative sections of irradiated lymph nodes. (A to D) Hematoxylin and eosin (HE) stains (top left) and immunohistochemistry, horseradish peroxidase method, hematoxylin counterstain. Bars = 250 µm. (A) Sham-irradiated DLN of the "TM IR + ICI" group on day 2 after tumor IR. Normal lymph node with distinct, moderately sized primary follicles (HE: asterisks: primary follicle, arrow: sinus at hilus), with normal distribution of vessels in cortex, medulla and sinuses (CD31: arrowheads). Follicles are cell rich (CD45R/B220: arrows), and there are moderately cellular T cell zones (CD3: arrow). Sinuses contain a low number of macrophages (Iba1: arrow: sinus at hilus). There are a few random apoptotic lymphocytes (caspase 3: arrowheads). (B) Irradiated DLN of the "TM + C-DLN IR + ICI" group on day 2 after tumor IR. The architecture is preserved (HE: arrows: sinuses), with normal distribution of vessels in cortex, medulla and sinuses (CD31: arrowheads). Follicles are small and moderately cellular (CD45R/B220: arrows), and there are moderately cellular T cell zones (CD3: arrow). Sinuses contain moderate numbers of macrophages (Iba1: arrows). There are a few random apoptotic lymphocytes (caspase 3: arrowheads). (C) Sham-irradiated DLN of the "TM IR + ICI" group on day 7 after tumor IR. Normal lymph node with distinct, moderately sized primary follicles (HE: asterisks), with normal distribution of vessels in cortex, medulla and sinuses (CD31: arrowheads). Follicles are cell rich (CD45R/B220: arrows), and there are relatively large T cell zones (CD3: asterisk). Sinuses contain a low number of macrophages (Iba1: arrow: sinus at hilus). There are a few random apoptotic lymphocytes (caspase 3: arrowheads). (D) Irradiated DLN of the "TM + C-DLN IR + ICI" group on day 7 after tumor IR. The architecture is preserved (HE: arrows: sinuses), with normal distribution of vessels in cortex, medulla and sinuses (CD31: arrowheads). Follicles are relatively small and moderately cellular (CD45R/B220: arrows), and there are moderately cellular T cell zones (CD3: asterisk). Sinuses contain moderate numbers of macrophages (Iba1: arrows). There are several individual and small groups of random apoptotic lymphocytes (caspase 3: arrowheads). Representative sections from n=3 mice per treatment group, 2 lymph nodes per mouse.

Supplementary Figure 6

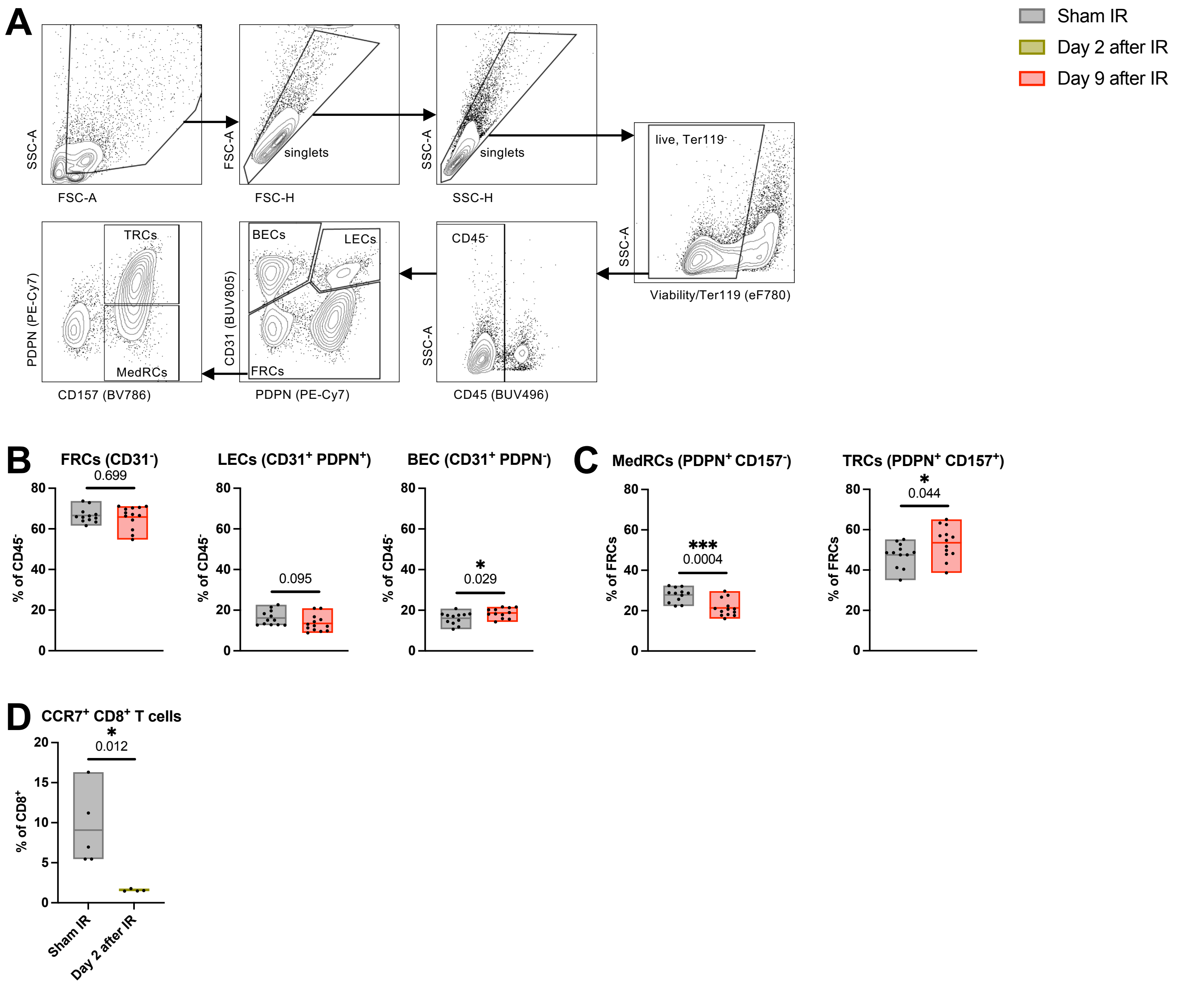

Supplementary Figure 6. Irradiation induces changes in the stromal cell compartment of the lymph node and interferes with the CCR7-CCL19/CCL21 immune cell homing axis. (A) Representative flow cytometry gating strategy for the analysis of lymph node stromal cells. Corresponding data is shown in Figure 8, C to E, and Supplementary Figure 6, B and C. **(B to D)** Lymph node stromal cells in response to IR, as described in Figure 8. Each dot represents an individual mouse. Floating bars span from the minimal to the maximal value of each group. Line indicates the mean. n=12 mice for the sham-irradiated group (gray) and n=13 mice for the group which received lymph node IR 9 days prior to analysis (red). (B) Percentages of fibroblastic reticular cells (FRCs), lymphatic endothelial cells (LECs) and blood endothelial cells (BECs) within the $CD45^-$ compartment. Populations are defined by differential expression of CD31 and podoplanin (PDPN). (C) Percentages of medullary reticular cells (MedRCs) and T zone reticular cells (TRCs) within the FRC compartment. (D) Percentage of $CCR7^+$ $CD8^+$ T cells within the $CD8^+$ T cell compartment. n=5 mice per group. Data were tested for normality using the Shapiro-Wilk test. For data following a normal distribution, treatment groups were compared using the two-sided unpaired t test (B, LECs and BECs, C and D). For non-normally distributed data, the comparison was performed using the two-sided Mann-Whitney test (B). All p values are displayed, with *, ** and *** indicating $p<0.05$, $p<0.01$ and $p<0.001$, respectively.

Supplementary Figure 7

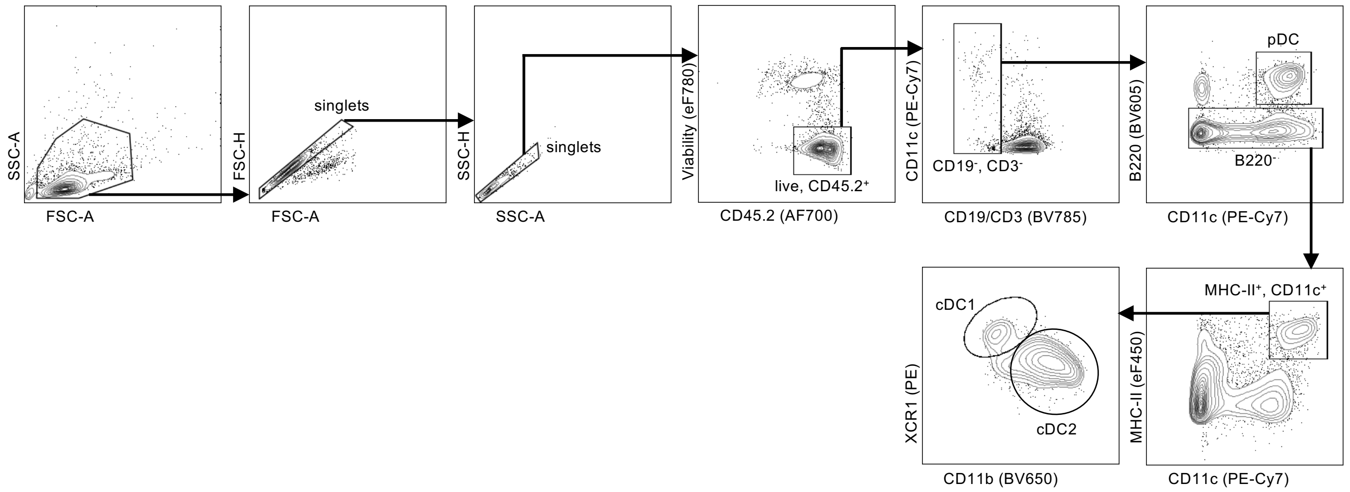

Supplementary Figure 7. Representative flow cytometry gating strategy for the analysis of DCs. Corresponding data is shown in Figure 10, C and D.

Supplementary Table 1. Antibodies used for flow cytometry and fluorescence activated cell sorting.

Target	Clone	Fluorochrome	Dilution	Reference	Distributor
B220	RA3-6B2	BV605	200	103243	Biolegend
CCR7	4B12	PE	50	120105	Biolegend
CD103	M290	BUV805	200	741948	BD Biosciences
CD103	M290	BUV395	200	740238	BD Biosciences
CD106 (VCAM-1)	429	BV711	400	740675	BD Biosciences
CD11b	M1/70	BV650	200	101259	Biolegend
CD11b	M1/70	BUV661	200	612977	BD Biosciences
CD11c	N418	PE-Cy7	200	117318	Biolegend
CD11c	N418	BV570	200	117331	Biolegend
CD152 (CTLA-4)	UC10-4B9	PE-Dazzle594	200	106318	Biolegend
CD157	BP-3	BV786	200	741012	BD Biosciences
CD157	BP-3	APC	200	140208	Biolegend
CD19	6D5	BV785	200	115543	Biolegend
CD19	6D5	AF647	200	115522	Biolegend
CD21/35	7E9	APC	200	123412	Biolegend
CD3	145-2C11	BV785	200	100355	Biolegend
CD31	MEC13.3	BUV805	200	741939	BD Biosciences
CD31	390	PerCP-Cy5.5	200	102420	Biolegend
CD39	24DMS1	PerCP-eF710	200	46-0391-82	eBioscience
CD4	GK1.5	BUV395	200	565974	BD Biosciences
CD4	GK1.5	PE-Cy7	200	100421	Biolegend
CD40	1C10	PE-Cy5	200	15-0401-82	eBioscience
CD44	IM7	BV711	200	103057	Biolegend
CD44	IM7	PE	200	103008	Biolegend
CD45	30-F11	BUV496	200	749889	BD Biosciences
CD45	30-F11	APC-Cy7	200	103116	Biolegend
CD45.2	104	AF700	200	109822	Biolegend
CD45.2	104	BUV737	200	612778	BD Biosciences
CD54 (ICAM-1)	3E2	BV421	200	564704	BD Biosciences
CD80	16-10A1	FITC	200	104705	Biolegend
CD86	GL-1	BV510	200	105039	Biolegend
CD8α	53-6.7	BUV395	200	563786	BD Biosciences
CD8α	KT15	Pacific Blue	200	MCA609PB	BioRad
CD8β	YTS156.7.7	APC	200	126614	Biolegend
FOXP3	FJK-16s	eF660	200	50-5773-82	eBioscience
FOXP3	FJK-16s	AF700	200	56-5773-82	eBioscience

Granzyme B	GB11	Pacific Blue	200	515408	Biolegend
IFNγ	XMG1.2	BV711	200	505836	Biolegend
Ki67	11F6	AF488	200	151204	Biolegend
Ki67	16A8	BV605	200	652413	Biolegend
Ly6A/E (Sca1)	D7	BV510	400	108129	Biolegend
Ly6C	AL-21	PerCP-Cy5.5	200	560525	BD Biosciences
MAdCAM-1	MECA-367	BUV615	200	751510	BD Biosciences
MHC-I	24-14-8	APC	200	17-5999-82	eBioscience
MHC-II (I/A-I/E)	M5/114.15.2	eF450	200	48-5321-80	eBioscience
MHC-II (I/A-I/E)	M5/114.15.2	BV480	200	566088	BD Biosciences
NK1.1	PK136	BV421	200	108732	Biolegend
PD-1	29F.1A12	BV510	200	135241	Biolegend
PDPN	36899	PE-Cy7	800	127412	Biolegend
TCF-1	S33-966	PE	200	564217	BD Biosciences
TCRβ	H57-597	BV785	200	109249	Biolegend
TER119	TER-119	APC-Cy7	200	116223	Biolegend
TIM-3	RMT3-23	PE-Cy7	200	119716	Biolegend
TNFα	MP6-XT22	BV650	200	506333	Biolegend
XCR1	ZET	PE	200	148203	Biolegend
XCR1	ZET	BV650	200	148220	Biolegend
Rat IgG2a, κ (isotype control)	RTK2758	PE	50	400507	Biolegend
CD16/32 (Fc block)	93	-	100	101302	Biolegend
Fixable Viability dye eF780			1000	65-0865-18	eBioscience
Fixable Viability dye Zombie NIR			500	423106	Biolegend

Supplementary Table 2. Antibodies used for immunofluorescence.

Target	Clone	Fluorochrome	Dilution	Reference	Distributor
aSMA	1A4	Cy3	1000	C6198	Sigma-Aldrich
B220	RA3-6B2	eF450	200	48-0452-82	eBioscience
CCL19	polyclonal	-	150	BAF880	R&D Systems
CD31	MEC13.3	AF594	400	102520	Biolegend
CD4	RM4-5	-	300	100508	Biolegend
F4/80	BM8	AF647	200	123122	Biolegend
LYVE1	ALY-7	eF660	200	50-0443-82	eBioscience
PDPN	8.1.1	-	300	127402	Biolegend
Anti-Syrian hamster		AF488	1000	107-545-142	Jackson ImmunoResearch
Anti-Syrian hamster		AF594	1000	307-585-003	Jackson ImmunoResearch
Streptavidin		AF647	1000	016-600-984	Jackson ImmunoResearch
