# Supplementary material for: Delayed tumor-draining lymph node irradiation preserves the efficacy of combined radiotherapy and immune checkpoint blockade in models of metastatic disease
Source: Nat Commun. 2024 Jun 29;15:5500. doi: 10.1038/s41467-024-49873-y (PMC11217506; doi:10.1038/s41467-024-49873-y)
Supplement: Supplementary file 3 — Reporting Summary [file 41467_2024_49873_MOESM3_ESM.pdf]

nature portfolio

Corresponding author(s): Prof. Dr. Martin Pruschy

Last updated by author(s): Jun 10, 2024

Reporting Summary

Nature Portfolio wishes to improve the reproducibility of the work that we publish. This form provides structure for consistency and transparency in reporting. For further information on Nature Portfolio policies, see our Editorial Policies and the Editorial Policy Checklist.

Statistics

For all statistical analyses, confirm that the following items are present in the figure legend, table legend, main text, or Methods section.

n/a	Confirmed	
☐	☒	The exact sample size (*n*) for each experimental group/condition, given as a discrete number and unit of measurement
☐	☒	A statement on whether measurements were taken from distinct samples or whether the same sample was measured repeatedly
☐	☒	The statistical test(s) used AND whether they are one- or two-sided<br>*Only common tests should be described solely by name; describe more complex techniques in the Methods section.*
☐	☒	A description of all covariates tested
☐	☒	A description of any assumptions or corrections, such as tests of normality and adjustment for multiple comparisons
☐	☒	A full description of the statistical parameters including central tendency (e.g. means) or other basic estimates (e.g. regression coefficient) AND variation (e.g. standard deviation) or associated estimates of uncertainty (e.g. confidence intervals)
☐	☒	For null hypothesis testing, the test statistic (e.g. *F*, *t*, *r*) with confidence intervals, effect sizes, degrees of freedom and *P* value noted<br>*Give P values as exact values whenever suitable.*
☒	☐	For Bayesian analysis, information on the choice of priors and Markov chain Monte Carlo settings
☒	☐	For hierarchical and complex designs, identification of the appropriate level for tests and full reporting of outcomes
☒	☐	Estimates of effect sizes (e.g. Cohen's *d*, Pearson's *r*), indicating how they were calculated

Our web collection on statistics for biologists contains articles on many of the points above.

Software and code

Policy information about availability of computer code

Data collection	Living Image v4.7.1, SmART-ATP, SpectroFlo v.3.0, BD FACSDiva v8.0.1, BD FACSChorus v1.3, QuantStudio Design & Analysis v.1.5.1, ZEN v14.0.18.201
Data analysis	Living Image v4.7.1, FlowJo v10.8, Imaris v9.2.1, QuPath v.0.3.2, Prism v9.5, Python v3.7

For manuscripts utilizing custom algorithms or software that are central to the research but not yet described in published literature, software must be made available to editors and reviewers. We strongly encourage code deposition in a community repository (e.g. GitHub). See the Nature Portfolio guidelines for submitting code & software for further information.

Data

Policy information about availability of data

All manuscripts must include a data availability statement. This statement should provide the following information, where applicable:

- Accession codes, unique identifiers, or web links for publicly available datasets
- A description of any restrictions on data availability
- For clinical datasets or third party data, please ensure that the statement adheres to our policy

The authors declare that all relevant data supporting the findings of this study are available within the paper and the Supplementary Information file. Source data are provided with this paper.

Research involving human participants, their data, or biological material

Policy information about studies with human participants or human data. See also policy information about sex, gender (identity/presentation), and sexual orientation and race, ethnicity and racism.

Reporting on sex and gender	Research did not involve human participants, their data, or biological material.
Reporting on race, ethnicity, or other socially relevant groupings	Research did not involve human participants, their data, or biological material.
Population characteristics	Research did not involve human participants, their data, or biological material.
Recruitment	Research did not involve human participants, their data, or biological material.
Ethics oversight	Research did not involve human participants, their data, or biological material.

Note that full information on the approval of the study protocol must also be provided in the manuscript.

Field-specific reporting

Please select the one below that is the best fit for your research. If you are not sure, read the appropriate sections before making your selection.

☒ Life sciences ☐ Behavioural & social sciences ☐ Ecological, evolutionary & environmental sciences

For a reference copy of the document with all sections, see nature.com/documents/nr-reporting-summary-flat.pdf

Life sciences study design

All studies must disclose on these points even when the disclosure is negative.

Sample size	Minimal sample size was determined based on pilot studies, which were performed to estimate the effect size (change in the absolute volume on day 30 for efficacy studies and change in the percentage of tumor-inflitrating CD8+ T cells for mechanistic studies) and the dropout rate due to tumor cell rejection, spontaneous regression of the tumor or development of necrosis before reaching the endpoint.
Data exclusions	Mice were excluded from the experiment due to the following reasons, defined prior to performing the experiments: tumor cell rejection, spontaneous regression of the tumor or development of necrosis within 1 week after tumor irradiation. Outliers were identified using the Grubbs' test and excluded accordingly.
Replication	Unless otherwise specified, all data are pooled or representative from a minimum of two independent experiments.
Randomization	Stratified randomization based on the tumor volume on a predetermined day after tumor injection was used for the treatment group assignment.
Blinding	Investigators were blinded to group allocation during all experimental procedures and data analyses.

Reporting for specific materials, systems and methods

We require information from authors about some types of materials, experimental systems and methods used in many studies. Here, indicate whether each material, system or method listed is relevant to your study. If you are not sure if a list item applies to your research, read the appropriate section before selecting a response.

Materials & experimental systems

n/a	Involved in the study	
☐	☒	Antibodies
☐	☒	Eukaryotic cell lines
☒	☐	Palaeontology and archaeology
☐	☒	Animals and other organisms
☒	☐	Clinical data
☒	☐	Dual use research of concern
☒	☐	Plants

Methods

n/a	Involved in the study	
☒	☐	ChIP-seq
☐	☒	Flow cytometry
☒	☐	MRI-based neuroimaging

Antibodies

Antibodies used	Flow cytometry and fluorescence activated cell sorting<br>Target,Clone,Fluorochrome,Dilution,Reference,Distributor

B220,RA3-6B2,BV605,200,103243,Biolegend
CCR7,4B12,PE,50,120105,Biolegend
CD103,M290,BUV805,200,741948,BD Biosciences
CD103,M290,BUV395,200,740238,BD Biosciences
CD106 (VCAM-1),429,BV711,400,740675,BD Biosciences
CD11b,M1/70,BV650,200,101259,Biolegend
CD11b,M1/70,BUV661,200,612977,BD Biosciences
CD11c,N418,PE-Cy7,200,117318,Biolegend
CD11c,N418,BV570,200,117331,Biolegend
CD152 (CTLA-4),UC10-4B9,PE-Dazzle594,200,106318,Biolegend
CD157,BP-3,BV786,200,741012,BD Biosciences
CD157,BP-3,APC,200,140208,Biolegend
CD19,6D5,BV785,200,115543,Biolegend
CD19,6D5,AF647,200,115522,Biolegend
CD21/35,7E9,APC,200,123412,Biolegend
CD3,145-2C11,BV785,200,100355,Biolegend
CD31,MEC13.3,BUV805,200,741939,BD Biosciences
CD31,390,PerCP-Cy5.5,200,102420,Biolegend
CD39,24DMS1,PerCP-eF710,200,46-0391-82,eBioscience
CD4,GK1.5,BUV395,200,565974,BD Biosciences
CD4,GK1.5,PE-Cy7,200,100421,Biolegend
CD40,1C10,PE-Cy5,200,15-0401-82,eBioscience
CD44,IM7,BV711,200,103057,Biolegend
CD44,IM7,PE,200,103008,Biolegend
CD45,30-F11,BUV496,200,749889,BD Biosciences
CD45,30-F11,APC-Cy7,200,103116,Biolegend
CD45.2,104,AF700,200,109822,Biolegend
CD45.2,104,BUV737,200,612778,BD Biosciences
CD54 (ICAM-1),3.00E+02,BV421,200,564704,BD Biosciences
CD80,16-10A1,FITC,200,104705,Biolegend
CD86,GL-1,BV510,200,105039,Biolegend
CD8a,53-6.7,BUV395,200,563786,BD Biosciences
CD8a,KT15,Pacific Blue,200,MCA609PB,BioRad
CD8b,YTS156.7.7,APC,200,126614,Biolegend
FOXP3,FJK-16s,eF660,200,50-5773-82,eBioscience
FOXP3,FJK-16s,AF700,200,56-5773-82,eBioscience
Granzyme B,GB11,Pacific Blue,200,515408,Biolegend
IFNg,XMG1.2,BV711,200,505836,Biolegend
Ki67,11F6,AF488,200,151204,Biolegend
Ki67,16A8,BV605,200,652413,Biolegend
Ly6A/E (Sca1),D7,BV510,400,108129,Biolegend
Ly6C,AL-21,PerCP-Cy5.5,200,560525,BD Biosciences
MAdCAM-1,MECA-367,BUV615,200,751510,BD Biosciences
MHC-I,24-14-8,APC,200,17-5999-82,eBioscience
MHC-II (I/A-I/E),M5/114.15.2,eF450,200,48-5321-80,eBioscience
MHC-II (I/A-I/E),M5/114.15.2,BV480,200,566088,BD Biosciences
NK1.1,PK136,BV421,200,108732,Biolegend
PD-1,29F.1A12,BV510,200,135241,Biolegend
PDPN,36899,PE-Cy7,800,127412,Biolegend
TCF-1,S33-966,PE,200,564217,BD Biosciences
TCRb,H57-597,BV785,200,109249,Biolegend
TER119,TER-119,APC-Cy7,200,116223,Biolegend
TIM-3,RMT3-23,PE-Cy7,200,119716,Biolegend
TNFa,MP6-XT22,BV650,200,506333,Biolegend
XCR1,ZET,PE,200,148203,Biolegend
XCR1,ZET,BV650,200,148220,Biolegend
Rat IgG2a, k (isotope control),RTK2758,PE,50,400507,Biolegend
CD16/32 (Fc block),93,-,100,101302,Biolegend
Fixable Viability dye eF780,-,-,1000,65-0865-18,eBioscience
Fixable Viability dye Zombie NIR,-,-,500,423106,Biolegend

Immunofluorescence
Target,Clone,Fluorochrome,Dilution,Reference,Distributor
aSMA,1A4,Cy3,1000,C6198,Sigma-Aldrich
B220,RA3-6B2,eF450,200,48-0452-82,eBioscience
CCL19,polyclonal,-,150,BAF880,R&D Systems
CD31,MEC13.3,AF594,400,102520,Biolegend
CD4,RM4-5,-,300,100508,Biolegend

	F4/80,BM8,AF647,200,123122,Biolegend<br>LYVE1,ALY-7,eF660,200,50-0443-82,eBioscience<br>PDPN,08.01.2001,-,300,127402,Biolegend<br>Anti-Syrian hamster,-,AF488,1000,107-545-142,Jackson ImmunoResearch<br>Anti-Syrian hamster,-,AF594,1000,307-585-003,Jackson ImmunoResearch<br>Streptavidin,-,AF647,1000,016-600-984,Jackson ImmunoResearch
Validation	All antibodies used in this study are commercially available and were validated by the manufacturers. Details are available on the manufacturers' websites.

Eukaryotic cell lines

Policy information about cell lines and Sex and Gender in Research

Cell line source(s)	The B16F10-Fluc-Puro murine melanoma cell line was purchased from Imanis Life Sciences (catalog number: CL052). The MC38 murine colorectal cancer cell line and the B16F10 murine melanoma cell line were a kind gift from Lubor Borsig (Dept. Physiology, University of Zurich, Switzerland).
Authentication	Cell lines were authenticated by the providers and used within 5 passages and 2 weeks after thawing for all experiments.
Mycoplasma contamination	All cell lines have tested negative for mycoplasma contamination.
Commonly misidentified lines (See ICLAC register)	We did not use any commonly misidentified cell lines.

Animals and other research organisms

Policy information about studies involving animals; ARRIVE guidelines recommended for reporting animal research, and Sex and Gender in Research

Laboratory animals	We used 7- to 8-week old female C57BL/6J mice purchased from Envigo (C57BL/6OlaHsd) or Janvier (C57BL/6JRj) for all experimental procedures.
Wild animals	The study did not involve wild animals.
Reporting on sex	Sex was not considered in this study.
Field-collected samples	The study did not involve field-collected samples.
Ethics oversight	All animal experiments were performed in accordance with the Swiss federal and cantonal laws on animal welfare and approved by the Cantonal Veterinary Office Zurich (ZH113/2020 and ZH141/2023).

Note that full information on the approval of the study protocol must also be provided in the manuscript.

Plants

Seed stocks	Plants were not used in this study.
Novel plant genotypes	Plants were not used in this study.
Authentication	Plants were not used in this study.

Flow Cytometry

Plots

Confirm that:

- [x] The axis labels state the marker and fluorochrome used (e.g. CD4-FITC).
- [ ] The axis scales are clearly visible. Include numbers along axes only for bottom left plot of group (a 'group' is an analysis of identical markers).
- [x] All plots are contour plots with outliers or pseudocolor plots.
- [x] A numerical value for number of cells or percentage (with statistics) is provided.

Methodology

Sample preparation	For immune cell analysis, tumors, brachial, axillary and inguinal lymph nodes were harvested and kept on ice in the flow cytometry buffer (PBS with 2% FBS and 2 mM EDTA) until processing. Tumors were cut into small pieces using scissors and incubated in an orbital shaker in 2 mL/sample of dissociation buffer [DMEM with 10% FBS, 0.5 mg/mL DNase I (Roche) and 1 mg/mL collagenase D (Roche)] for 45 minutes at 37C and 100 rpm. Lymph nodes were disrupted into small pieces using two 26G needles and digested in 500 uL/sample of dissociation buffer for 15 minutes at 37C and 100 rpm. Following digestion, all organs were passed through a 70 um cell strainer using a syringe plunger. Absolute cell counts were obtained from the single cell suspension using the EVE automatic cell counter (NanoEntek). For the IFNg and TNFa staining, single cell suspensions were first stimulated ex vivo for 3.5 hours at 37C in 200 uL/sample of activation buffer [DMEM with 10% FBS, 100 ng/mL phorbol 12-myristate 13-acetate (PMA, Sigma-Aldrich), 1 ug/mL ionomycin (Sigma-Aldrich) and 5 ug/mL brefeldin A (eBioscience)]. Cells were incubated in the extracellular staining mix for 30 minutes at 4C, followed by fixation and permeabilization using the Foxp3/Transcription factor staining buffer set (eBioscience) according to the manufacturer's instructions. Intracellular staining was performed overnight.<br>For stromal cell analysis and fluorescence activated cell sorting, brachial, axillary, and inguinal lymph nodes were harvested and kept on ice in Roswell Park Memorial Institute 1640 medium (RPMI 1640, Gibco) supplemented with 2% FBS until processing. Stromal cell isolation was initiated by disrupting the lymph nodes into small pieces using two 26G needles and transferring the pieces into 2mL/sample of dissociation buffer [RPMI 1640 with 2% FBS, 20 mM 4-(2-hydroxyethyl)-1-piperazineethanesulfonic acid, pH 7.2 (HEPES, Lonza), 200 ug/mL collagenase P (Roche), 30 ug/mL dispase (Roche) and 10 ug/mL DNase I (Roche)]. Tissues were subsequently incubated for 45 minutes at 37C, with resuspension and collection of the supernatant every 15 minutes. Stromal cells were enriched by incubating the cell suspensions with magnetic anti-CD45 and anti-TER119 beads (MACS MicroBeads, Miltenyi Biotec) for 20 minutes at 4C, followed by passing the suspensions through MACS LS columns (Miltenyi Biotec). Unbound single cell suspensions were subsequently incubated for 20 minutes at 4C with the viability dye eFluor 780 (Invitrogen) and for 30 minutes at 4C with the respective antibodies.
Instrument	Cytek Aurora (5 laser), FACSymphony, FACSMelody
Software	Data was collected using SpectroFlo v.3.0, BD FACSDiva v8.0.1 and BD FACSChorus v1.3, and analyzed using FlowJo v10.8.
Cell population abundance	The abundance of the relevant cell populations was confirmed by flow cytometry using FACSymphony (BD Biosciences). The purity of the sorted cell fraction was determined using the sorting statistics generated by the FACSChorus software with a sorting efficiency of over 95%.
Gating strategy	Singlets were gated using FSC-A/FSC-H and SSC-A/SSC-H plots. Viable cells were determined using fixable viability dyes (eF780 and Zombie NIR), followed by gating for CD45 positive (immune cells) or negative (stromal cells) populations. Further populations were defined as illustrated in supplementary figures: see Supplementary Fig. 1A for tumor lymphocytes gating strategy, Supplementary Fig. 4A for lymph node lymphocytes gating strategy, Supplementary Fig. 6A for lymph node stromal cell gating strategy and Supplementary Fig. 7 for lymph node dendritic cells gating strategy. FMO controls were used for markers without a distinct negative and positive population.

☒ Tick this box to confirm that a figure exemplifying the gating strategy is provided in the Supplementary Information.
